# Friedelin: A natural compound exhibited potent antibacterial, anti-inflammatory, and wound healing properties against MRSA-infected wounds

**DOI:** 10.1007/s00210-025-03965-8

**Published:** 2025-03-18

**Authors:** Riham A. El-Shiekh, Mai Hussin Radi, Rana Elshimy, Essam Abdel-Sattar, Ali M. El-Halawany, Marwa A. Ibrahim, Merhan E. Ali, Eman I. Hassanen

**Affiliations:** 1 Department of Pharmacognosy, Faculty of Pharmacy, Cairo University, Cairo, 11562 Egypt; 2 Herbal Department, Egyptian Drug Authority, Cairo, 15301 Egypt; 3 Department of Microbiology and Immunology, Faculty of Pharmacy, Ahram Canadian University, Giza, 12573 Egypt; 4 Department of Microbiology and Immunology, Egyptian Drug Authority, Cairo, 15301 Egypt; 5 Department of Biochemistry and Molecular Biology, Faculty of Veterinary Medicine, Cairo University, Giza, 12211 Egypt; 6 Department of Pathology, Faculty of Veterinary Medicine, Cairo University, Giza, 12211 Egypt

**Keywords:** Friedelin, Gene expression, MRSA, Pathology, Wound healing

## Abstract

Methicillin-resistant Staphylococcus aureus (MRSA) is primarily recognized as a pathogen responsible for skin, soft tissue, and multiple organs infection. The colonization of the skin and mucous membranes by hypervirulent resistant bacteria like MRSA during hospitalization significantly contributes to life-threatening conditions. Friedelin (FRN) is a pentacyclic triterpene (C_30_H_50_O) isolated from *Euphorbia grantii* Oliv. The current work aims to determine the efficacy of FRN against MRSA-infected wounds in mice besides the in vitro study to evaluate its bactericidal activity. The in vitro study revealed that FRN was strongly active against MRSA which had a wide zone of MRSA growth inhibition and promising minimum inhibitory concentration (MIC). Moreover, FRN downregulated the major virulence genes *seb* and *icaD*, responsible for the production of staphylococcal enterotoxin SED and biofilm formation, respectively in contrast to the untreated group. The dressing of MRSA-infected wound with 40 ppm FRN significantly reduced the wound size and bacterial count and accelerated the process of wound healing which had a higher immune expression of both VEGF (vascular endothelial growth factor) and α-SMA (alpha smooth muscle actin) compared with other treated groups. Additionally, FRN could reduce the inflammatory response of MRSA in a dose-dependent manner by downregulating the *TNF-α (tumor necrosis factor-α)* and *PGS-2 (prostaglandin synthase-2*) gene expression levels. FRN is effective against MRSA-infected wounds via its potent bactericidal and anti-inflammatory activities that accelerate angiogenesis and wound maturation.

**Graphical Abstract:**

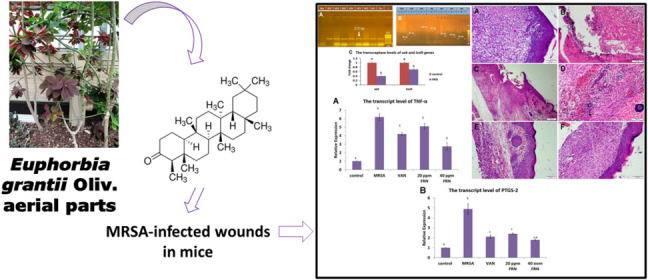

**Supplementary Information:**

The online version contains supplementary material available at 10.1007/s00210-025-03965-8.

## Background

Skin is the most vulnerable to external invasion, and many factors will delay wound healing like diabetes, infections, and pressure ulcers resulting in chronic wounds (Wysocki 2002). Therefore, chronic wound treatment and care has developed an urgent demand in clinics (Li et al. 2022). Chronic wounds are regularly invaded by foreign bodies and bacteria resulting in wound infection and delay of its repair (Daeschlein 2013). Moreover, multidrug resistance of bacteria has proposed a progressively serious threat to human health invalidating the primary therapeutic mode of using traditional antibiotics (Ouyang et al. 2018). One of the most hazardous pathogens is MRSA, which is not sensitive to most of the traditional antibiotics and seriously warns public health (Huh and Kwon 2011; Zhong et al. 2020). Despite being considered the only effective antibiotic against MRSA infections during the last six decades, vancomycin treatment failure is not egregious even if the isolates showed total susceptibility towards this antibiotic. Nephrotoxicity and restricted careful monitoring of vancomycin levels are the most common drawbacks associated with its use against MRSA (Sharma and Hammerschlag 2019).

Furthermore, vancomycin-resistant bacteria such as vancomycin-resistant *Staphylococcus aureus*, vancomycin-intermediate sensitive staph, and heterogeneous vancomycin-intermediate *S. aureus* are considered a worrying obstacle facing health care providers and a critical alarm that makes finding an alternative treatment an urgent need (Elnour and Ramadan 2021). The most effective strategy to tackle this significant issue is to develop new antibiotics. Unfortunately, the rate of new antibiotic development falls far behind the rate of bacterial resistance development (Zhong et al. 2020).

Recently, various herbal medications and formulations have been widely used for their new antibacterial and wound healing capabilities, including alkaloids, tannins, flavonoids, and terpenes (Somwong et al. 2022; Agra et al. 2015). The high prevalence of severe bacterial and viral infections, with their tendency to become resistant against conventional antibiotics, emphasizes the urgent concerns to find novel compounds to combat them. Friedelin (FRN, Fig. S1) ((4*R*,4*aS*,6*aS*,6*aS*,6*bR*,8*aR*,12*aR*,14*aS*,14*bS*)−4,4*a*,6*a*,6*b*,8*a*,11,11,14*a*-octamethyl-2,4,5,6,6*a*,7,8,9,10,12,12*a*,13,14,14*b*tetradecahydro-1*H*-picen-3-one) could be used because of its low cytotoxicity and powerful pharmacological activities (Radi et al. 2023a). FRN is a pentacyclic triterpene isolated from various plant families such as Euphorbiaceae, Fagaceae, Rosacea, and Asteraceae. It is recognized to have a variety of pharmacological actions including anti-inflammatory, antibacterial, and antiviral properties (Saber et al. 2024; Radi et al. 2023b, 2023c; Radi et al. 2023e). Acute toxicity studies in mice demonstrated the safety of this substance at doses ranging from 300- to 700-mg/kg body weight (Duraipandiyan et al. 2016). However, other investigations revealed that the most effective nontoxic dose of FRN in mice is up to 80 mg/kg body weight (Shi et al. 2021). The in vitro antibacterial activity of FRN was investigated against several strains of Gram-positive and Gram-negative bacteria, where the compound displayed a wide range of antibacterial activity with MIC values ranging from 2.44 to 78.12 μg/mL when compared to the other compounds and standard antibiotics (Radi et al. 2023c).

Recent studies, including in vitro and in vivo investigations, have found that triterpenes significantly accelerate wound healing by increasing epithelialization and collagen production and deposition, regardless of wound type. Furthermore, their inclusion into various medical formulations offers a useful option for long-term distribution of active phytochemicals, making them a promising developing class of medicines in the treatment and management of wounds (Agra et al. 2015; Ghiulai et al. 2020; Ticona et al. 2022). Friedelin’s wound healing activity was previously examined in vitro in keratinocytes. FRN was found to drastically reduce wound area and improve keratinocyte migration by increasing matrix metalloproteinase-9 synthesis and keratin-17, whereas the mRNA gene expressions of *cadherin-1*, *desmoglein-1*, inflammatory genes (*Cox-2* and *iNOS*), and pro-inflammatory cytokine genes (*TNF-α* and *IL-6*) were significantly decreased (Somwong et al. 2022).

In the current study, we planned to evaluate the possible bactericidal and wound healing action of FRN, which had previously been identified as the main component from *Euphorbia grantii* Oliv. (Radi et al. 2023d) with the explanation of its molecular mechanisms to provide useful information for expediting wound healing and inspiring combat against MRSA-infected wounds.

## Methods

See Supplementary file.

## Results

### Multiplex PCR and quantitative RT-PCR analysis of virulence genes

**Fig. 1:**
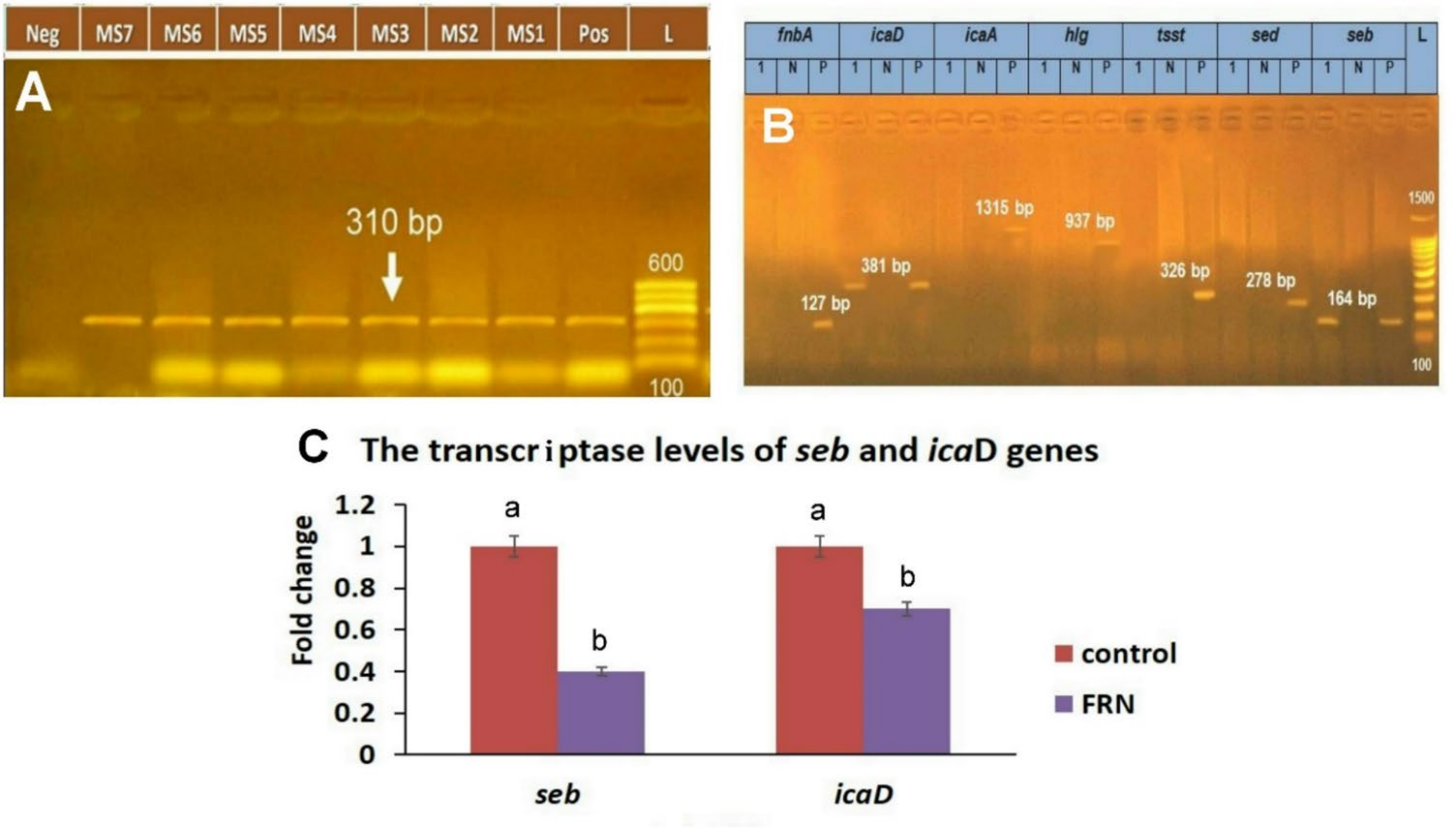
**A** Agarose gel photo electrophoresis for the PCR products of the *mec*A gene of the MRSA isolates. L: Lane, Lane Pos: positive control, Lane MS1-MS7: MRSA isolates carrying *mec*A gene with an amplicon size of 310 bp. Lane Neg: negative control (Rana et al. 2018). **B** Agarose gel electrophoresis for the PCR products of the virulence and resistance genes performed on the MRSA isolates. *fnbA*, fibronectin-binding protein A; *icaD,* inhibitor of caspase-activated deoxyribonuclease; *icaA*, intercellular adhesion A; *Hlg*, gamma-hemolysin; *Tsst*, toxic shock syndrome toxin-1; *sed*, staphylococcal enterotoxin D; and *seb*, staphylococcal enterotoxin B. The isolate was positive for *seb* and *icaD* genes and showed two bands at 164 bp and 381 bp, respectively. **C** Bar chart representing the fold change of *icaD* and *seb* expression upon FRN treatment. Values are presented as mean ± SEM. Different letters (a, b) indicate a significant difference at *P* ≤ 0.05

The MRSA isolate was positive for the major virulence genes *seb* and *icaD* that showed two bands at 164 bp and 381 bp, respectively (Fig. 1A and B). Otherwise, FRN effectively downregulated *seb* and *icaD* to 0.4 and 0.71, respectively **(**Fig. 1C).

### In vitro antimicrobial efficacy of FRN

Our results showed a wide zone of MRSA growth inhibition surrounding discs of VAN and FRN, while the plates incubated with either DMSO or Amikacin did not record any inhibitory zone. Regarding MIC, the lowest concentration was recorded in VAN-incubated plate followed by FRN (Table 1).

**Table 1:** Inhibition zone (IZ) and (Minimum inhibitory concentration) MIC of antibiotics and FRN on Methicillin-resistant *Staphylococcus aureus* (MRSA)

	DMSO	AMK	VAN	FRN
IZ (mm)	0	0	19.3 ± 0.3	17.3 ± 1.2
MIC (μg/mL)	-	-	2 ± 0	13.3 ± 2.7

Data expressed as *mean* ± *SEM*

### Wound contraction

The rate of wound contraction was markedly increased in all treated groups compared with the MRSA-infected untreated group. Comparing treatment groups, the group treated with 40 ppm FRN showed a higher percentage of wound contraction than other treated groups, even exceeding the rate of non-infected control group. Otherwise, MRSA-infected non-treated wound recorded the lowest wound contraction rate (Table 2).

**Table 2:** Wound contraction ratio (%) in different treatment groups

Interval	Non-infected	MRSA-infected	MRSA-infected + VAN	MRSA-infected + 20 ppm FRN	MRSA-infected + 40 ppm FRN
3 days	35 ± 1.3^b^	1.5 ± 0.16^a^	34.65 ± 1.5^b^	38.46 ± 1.3^b^	46.15 ± 0.5^c^
7 days	60 ± 0.9^c^	7.69 ± 0.8^a^	53.85 ± 1.05^b^	51.92 ± 0.38^b^	65 ± 0.88^c^
10 days	80 ± 1.4^c^	15.38 ± 0.5^a^	62.69 ± 0.7^b^	69.23 ± 1.1^b^	86.5 ± 0.8^c^

Data expressed as *mean* ± *SEM* (*n* = 7); different superscript letters (a, b, and c) indicate significance between groups at *P* ≤ 0.05

### Total bacterial count

At 0-day post-inoculation, the bacterial colonies had the same count in all groups. Otherwise, FRN had the ability to reduce the bacterial count in contrast to the MRSA-infected group in dose-dependent manner at 3, 7, and 10 days. The lowest bacterial count was recorded in 40 ppm FRN-treated groups at 7 days, whereas the total bacterial clearance was noticed at 10 days **(**Table 3).

**Table 3:** Quantification of bacterial loads of MRSA at different time points (CFU/g)

	Non-infected	MRSA-infected	MRSA-infected + VAN	MRSA-infected + 20 ppm FRN	MRSA-infected + 40 ppm FRN
0 day	< 100^b^	1.4 × 10^8^ ± 8.2 × 10^7a^	4.3 × 10^8^ ± 1.5 × 10^8a^	8.5 × 10^8^ ± 5.7 × 10^8a^	5.3 × 10^8^ ± 2.6 × 10^8a^
3 days	< 100^c^	1.8 × 10^8^ ± 1.6 × 10^8 a^	1.5 × 10^4^ ± 1.2 × 10^4b^	5.3 × 10^8^ ± 2.6 × 10^8a^	5.2 × 10^3^ ± 3.3 × 10^3b^
7 days	< 100^c^	2.7 × 10^7^ ± 2.7 × 10^6a^	1.5 × 10^3^ ± 7.8 × 10^2b^	1.6 × 10^3^ ± 9.9 × 10^2b^	3.1 × 10^2^ ± 1.6 × 10^2b^
10 days	< 100^c^	8.4 × 10^6^ ± 8.3 × 10^6 a^	3.7 × 10^2^ ± 1.4 × 10^2b^	3.3 × 10^2^ ± 9.5 × 10b	< 100 c

Data expressed as *mean* ± *SEM* (*n* = 7); different superscript letters (a, b, and c) indicate significance between all MRSA groups at *P* ≤ 0.05

### Quantitative RT-PCR analysis of PGS-2 and TNF-α genes

**Fig. 2:**
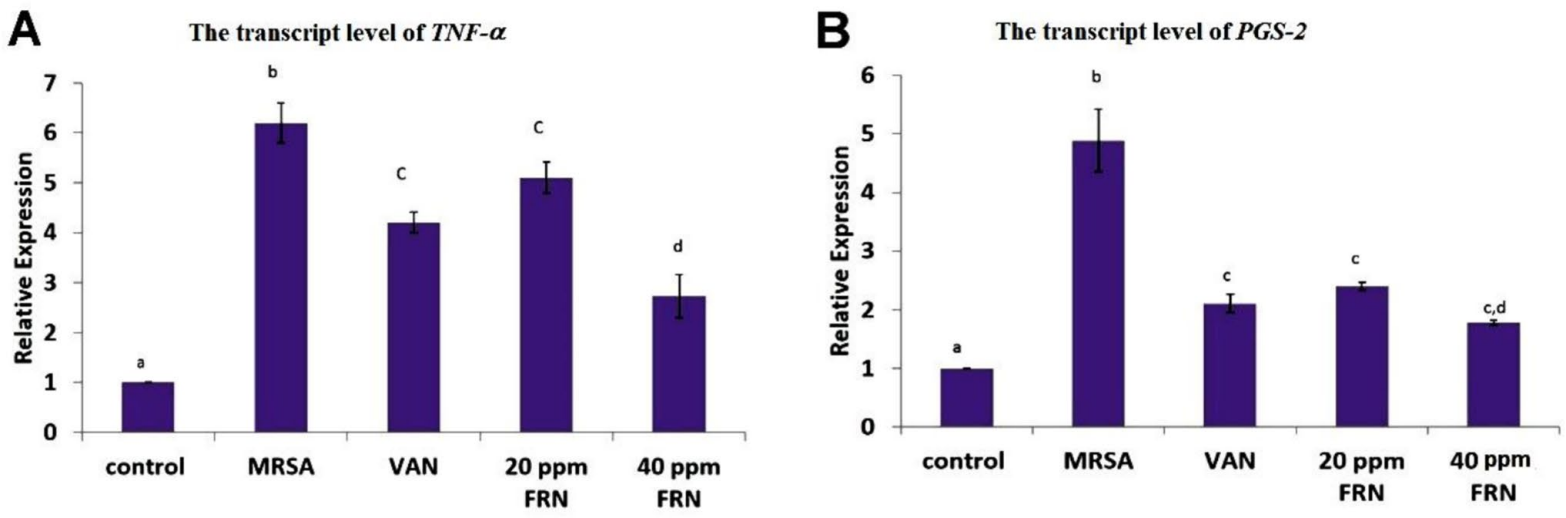
Bar chart represents the transcript levels of **A** *TNF-α* and **B** *PGS-2*. Values are presented as *mean* ± *SEM* (*n* = 7). Different superscript letters (a, b, c, and d) indicate a significant difference at *P* ≤ 0.05 (day 10)

In the current study, quantitative real-time PCR was done to assess the relative expression levels of both *TNF-α* and *PGS-2* in the skin of all experimental groups. The findings revealed that MRSA-infection significantly increased the studied genes. However, FRN treatments greatly ameliorated the adverse effects of the MRSA by downregulating the *TNF-α* and *PGS-2* genes in all treated groups, mainly the high dose FRN group (Fig. 2).

### Histopathology

**Fig. 3:**
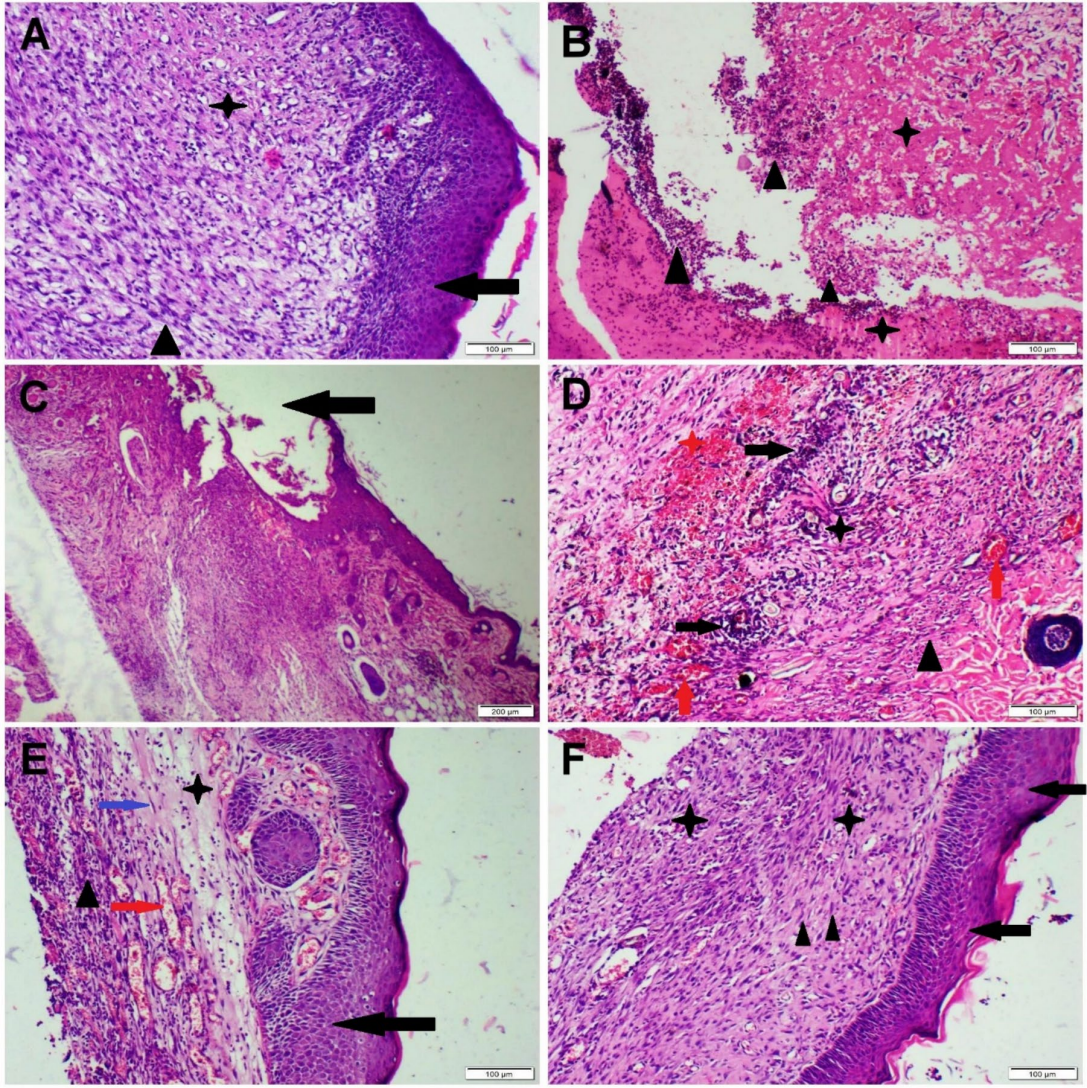
H&E-stained photomicrographs of skin tissue sections obtained from the wound area at 10 days that represent different treatment groups. **A** Non-infected mice showed complete re-epithelization (arrow), well-developed granulation tissue (star), and well-organized fibrous tissue (triangle). **B** and **C** MRSA-infected mice showed complete epidermal ulceration (arrow), extensive exudation (star), and neutrophilic infiltration (triangle). **D** VAN-treated mice with noticed prominent angiogenesis (red arrow), hemorrhage (red star), fibroplasia (black triangle), and moderate inflammatory cells’ infiltration (black arrow). **E** 20-ppm FRN-treated rat displayed complete re-epithelization (black arrow), prominent angiogenesis (red arrow), fibroplasia (black arrow), mild inflammatory cells (triangle), and exudations (blue arrow). **F** 40-ppm FRN-treated rat displayed complete re-epithelization (arrow) and thick granulation tissue (star) with regular collagen fiber deposition (triangle)

On day 10, the skin tissue sections obtained from the non-infected wound revealed fully formed epidermis with an enormous area of granulation tissue at the edge and bed of the wound (Fig. 3A). It has been packed with many fibroblasts, few collagens deposition, and obvious angiogenesis. On the other hand, the MRSA-infected wound displayed extensive deterioration in the process of wound healing. There was complete epidermal ulceration along with extensive neutrophilic infiltration (Fig. 3B). The dermis extensively infiltrated with neutrophils along with complete necrosis in the wound edge (Fig. 3C). Less vascular granulation tissue was detected in the wound bed of some sections. The group treated with VAN exhibited sensible improvement in the process of wound healing compared with the untreated MRSA-infected wound. There was incomplete re-epithelization with a moderate reduction in the inflammatory cells’ infiltration within the wound edge and bed **(**Fig. 3D). Moreover, a noticeable increase in the granulation tissue full of fibroblasts and prominent angiogenesis along with few collagen fibers was recorded in most sections. Conversely, remarkable improvement in the process of wound healing was noticed among FRN-treated groups in dose-dependent manner. The group treated with the low dose showed complete re-epithelization, moderate reduction of both inflammatory cells and exudations along with moderate collagen deposition (Fig. 3E). The granulation tissue contained many vessels and some cells with few fibers. Furthermore, the group treated with the high dose demonstrated complete re-epithelization and thick granulation tissue with regular collagen fiber deposition (Fig. 3F). The granulation tissue became more fibrous and less cellular with reduction in the microvascular density compared with other groups.

Based on the histological scoring system, all sections obtained from MRSA-infected wound demonstrated a slight degree of wound repair compared with the other groups. Otherwise, the VAN-treated group displayed moderate degree of wound healing, while FRN-treated groups showed marked degree to complete wound repair similar to those of the non-infected wound (Table 4).

**Table 4:** Histological lesion scoring of wounded areas in different treated groups

	Non-infected	MRSA-infected	MRSA-infected + VAN	MRSA-infected + 20 ppm FRN	MRSA-infected + 40 ppm FRN
Re-epithelization	+ + + +	-	+ +	+ + +	+ + + +
Congestion	+ +	+ + + +	+ + +	+ +	+
Exudation	-	+ + + +	+ +	+	-
Inflammatory cells	+ +	+ + + +	+ + +	+ +	+
Hemorrhage	-	+ + + +	+ +	-	-
Granulation tissue	+ + + +	+	+ + +	+ + + +	+ + + +
Angiogenesis	+ + + +	+	+ + +	+ + + +	+ + + +
Collagen	+ + + +	-	+ +	+ + +	+ + + +

### Immunohistochemistry

**Fig. 4:**
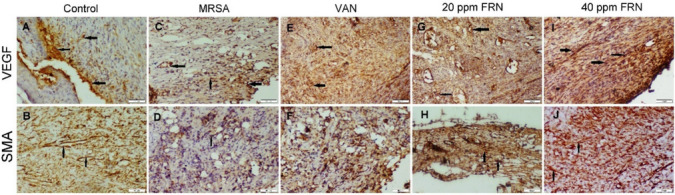
Photomicrographs representing α-SMA and VEGF immunostaining in various groups. **A** and **B** Non-infected mice showed moderate expression of VEGF and α-SMA, respectively. **C** and **D** MRSA-infected mice showed weak immunopositivity of VEGF and α-SMA, respectively. **E** and **F** VAN-treated mice noticed strong VEGF expression and moderate α-SMA immune expression, respectively. **G** and **H** 20-ppm FRN-treated mice displayed strong immunopositivity of both VEGF and α-SMA, respectively. **I** and **J** 40-ppm FRN-treated mice displayed strong immunopositivity of VEGF and α-SMA, respectively. (Arrows) indicate brown positive immune reactions

The non-infected wound displayed moderate VEGF and α-SMA immunopositivity. Otherwise, MRSA-infected wound displayed weak expression of both immune markers. A significant increase in the immunopositivity of both markers was detected in both FRN treated groups, but better improvement was noticed in FRN high dose-treated group; whereas, VAN exhibited lower levels of VEGF and α-SMA immune expression than FRN-treated groups but higher than MRSA-infected wound (Fig. 4 and Table 5).

**Table 5:** Immunohistochemical scoring in different experimental groups

	Non-infected	MRSA-infected	MRSA-infected + VAN	MRSA-infected + 20 ppm FRN	MRSA-infected + 40 ppm FRN
VEGF					
Mean % area score	2.5 ± 1^b^	1.5 ± 1^a^	3 ± 0.25^b^	3 ± 1^b^	4 ± 2^c^
Intensity score	2 ± 0^b^	1 ± 1^a^	3 ± 1^c^	3 ± 0^c^	3 ± 0^c^
Total score	4 ± 1^b^	3 ± 1^a^	6 ± 0.25^c^	6 ± 1^c^	7 ± 2^d^
α-SMA					
Mean % area score	3 ± 1^b^	1 ± 1^a^	3 ± 0.25^b^	3 ± 1^b^	5 ± 0^c^
Intensity score	3 ± 0^b^	1 ± 1^a^	3 ± 0^b^	3 ± 0^b^	3 ± 0^b^
Total score	6 ± 1^b^	2 ± 1^a^	6 ± 0.25^b^	6 ± 1^b^	8 ± 0^c^

Data expressed as medium ± *IQR* (*n* = 35); different superscript letters (a, b, and c) indicate significance between groups at *P* ≤ 0.05

## Discussion

Due to the elevating morbidity and mortality associated with hypervirulent MRSA infections especially for hospitalized patients’ wounds, treatment of this virulent and multidrug-resistant strains has become a major clinical priority (Wang et al. 2021). Wound healing is a complex process where several histological changes occur as re-epithelization, inflammation, granulation tissue, angiogenesis, and collagen deposition that finally leads to wound contraction (Gonzalez et al. 2016). Many factors can interfere with these processes such as bacterial infection and many metabolic disorders (Mendes et al. 2022). MRSA has emerged because of *S. aureus’* quick adaptation to selective antibiotics (Elnour and Ramadan 2021; Lowy 2003; Hassanen and Ragab 2021). So, it is important to find alternative antimicrobials based on naturally occurring compounds to overcome this serious health issue. Therefore, the current study aimed to investigate the role of FRN dressing to kill surface bacteria and improve wound healing process in MRSA-infected wound model via measuring the wound contraction, bacterial count, and histopathological scoring of the wounded area as well as exploring the effect of FRN on regulation of two major virulence MRSA genes. Additionally, we assessed the role of VEGF, α-SMA, TNF-α, and PGS-2 in such repair mechanisms.

The ability of MRSA to cause various serious infections in humans is attributed to the expression of virulence factors that cooperate in the infection pathogenesis by enhancing bacterial adhesion to the surfaces of host tissue, invasion, or avoidance of the immune system. Therefore, a successful replacement therapy must not only have a bactericidal or bacteriostatic effect but also should be able to suppress level of those attributes (Gharaibeh et al. 2020). Upon performing qRT-PCR in our study, FRN effectively downregulated the transcriptional level of MRSA enterotoxin producing gene *seb* and biofilm forming gene *icaD*. This agreed with Guzzo et al. who reported that nootkatone could down regulate the expression levels of *sarA*, *icaA*, *agrA*, *RNAIII*, and *spa* (Guzzo et al. 2020).

In the present study, the microscopic examination of the non-infected wound at 10 days showed normal process of wound repair along with normal baseline immune expression of both VEGF and α-SMA in the newly formed vascular capillaries and myofibroblasts, respectively. Otherwise, the MRSA-infected wound showed marked deterioration in these processes along with weak immune expression of both VEGF and α-SMA. It has been demonstrated that the vascular permeability increases in the initial stages of wound healing, enabling the deposition of fibrin-rich matrix required for cellular migration and proliferation (Wu et al. 2016). Furthermore, angiogenesis is essential for wound healing because it creates the vasculature that maintains the newly formed granulation tissue. Additionally, the pathway of wound healing involves both migration and activation of several cells like endothelium, activated fibroblasts (myofibroblasts), and various inflammatory cells. During healing, the activated fibroblasts turned into myofibroblasts expressing α-SMA (Klingberg et al. 2013). These cells produce an excessive extracellular matrix (ECM) that potentially causes a scar formation (Mokos et al. 2017). It is reported that several pro-inflammatory cytokines can inhibit myofibroblast differentiation that contribute to deterioration of wound repair mechanism (Larouche et al. 2018).

Among the main inflammatory cells invading the MRSA-infected wound are neutrophils and macrophages, which are the main sources of several growth factors and pro-inflammatory cytokines and mediators (Naldini and Carraro 2005; Hassanen et al. 2023a). The neutrophils can engulf and kill many bacteria; however, *S. aureus* fights neutrophils at the wound site either directly by leukotoxins that kill them or indirectly via secreting chemotaxis inhibitory protein that binds with C5a and formyl peptides interfering neutrophil chemotaxis (Iwatsuki et al. 2006; Haas et al. 2004). Moreover, it also reduces the bioavailability of VEGF in the wounded area via either producing various proteases as plasmin that degrade VEGF or decreasing the expression of VEGF receptor on the endothelial cells (Lauer et al. 2000; Power et al. 2001). During tissue injury, prostaglandins are formed by metabolism of arachidonic acid by cyclooxygenases (Cox) (Kapoor et al. 2007), and these prostaglandins play a key role in the generation of the inflammatory response (Ricciotti and FitzGerald 2011). Also, TNF-α plays a vital function in the healing process by regulating different cytokines and is used to evaluate the degree of inflammation during the wound repair process (Bakr et al. 2021). A reduction in TNF-α levels is necessary for quicker wound healing (Oliveira et al. 2016). Our findings revealed that MRSA upregulated the m-RNA levels of both inflammatory genes *PGS-2* and *TNF-α* in the wound area, indicating the incidence of progressive inflammatory reactions. It is well-known that wound bacterial infection enhance excessive inflammatory cell infiltration with subsequent release of several pro-inflammatory cytokines/mediators as TNF-α and PGS-2 (Power et al. 2001). We suggested the permanent elevation of *TNF-α* gene levels could delay the wound repair in MRSA-infected mice. Our suggestion may be agreed with (Pierce 2001) who found marked increase in pro-inflammatory cytokines such as interleukin-1β and TNF-α in non-healing diabetic wounds. Moreover, the activated neutrophils and macrophages released reactive oxygen species (ROS) resulting in necrosis of many cells in the wound area that contribute to delay the repair process (Khalaf et al. 2019). ROS can damage the membrane phospholipids releasing arachidonic acids that converted into prostaglandins via PGS-2 (Ebedy et al. 2022). The prostaglandins induce further inflammation via increasing the vascular permeability (Hassanen et al. 2023b). Numerous ligands can activate the *PGS-2* gene in a wide range of cells, including fibroblasts, macrophages, smooth muscle cells, and endothelial cells.

Friedelin extracted from different sources had been reported to have antifungal and antibacterial effects against wide range of Gram-positive pathogens such as *Bacillus cereus*, *Bacillus subtilis*, and *Staphylococcus aureus*, it can be used either alone, in combination with conventional antibiotics or as a resistance modulator (Gowdu Viswanathan et al. 2012; Catteau et al. 2018). Against MRSA, Odeh et al. reported a result of MIC of 10 μg/mL by friedelan-3-one (Odeh et al. 2016). In the current work, FRN at both high and low dosage levels significantly reduced the bacterial count at the wound site and improved the healing process compared with both untreated and VAN-treated groups. Despite growing studies on FRN’s medical applications, there is still a research gap about how FRN affects the process of wound healing. Additionally, there is a debate over the evidence indicating the antimicrobial effects of FRN, and all studies have focused on the drug’s in vitro bactericidal effects rather than its in vivo bactericidal effects. Several studies revealed the in vitro bactericidal effect of FRN against various Gram-positive and Gram-negative bacteria, which even exceeded the performance of the conventional antibiotic (Kamdem et al. 2022; Pretto et al. 2004). On the other hand, other studies showed that FRN had either poor or no antimicrobial activity against several Gram-positive and Gram-negative bacteria (Ogunnusi et al. 2010). We found that FRN increased the percentage of wound contraction, re-epithelization, and angiogenesis as well as reduced the inflammatory cells infiltration in the wound area. These findings are attributed to the potential anti-inflammatory, antibacterial, and antioxidant properties of FRN (Antonisamy et al. 2011; Sunil et al. 2013; Mokoka et al. 2013). FRN is a triterpenoid that has shown excellent biological properties (Antonisamy et al. 2015). Triterpenoids have attracted attention as wound healing agent (Razwinani and Motaung 2022). In the present study, FRN dose-dependently downregulated the transcription levels of both *TNF-α* and *PGS-2* proving its anti-inflammatory potential. Currently, the anti-inflammatory potential of FRN has been demonstrated in various animal models. Recent study confirmed the ability of FRN to reduce the pro-inflammatory cytokines in murine-ulcerative colitis model (Shi et al. 2021). Antonisamy and coworkers showed that FRN alleviates the local acute inflammatory reactions induced by both carrageenan and croton oil in mice (Antonisamy et al. 2011). Another recent study proved the anti-inflammatory and antioxidant effects of FRN via reducing many pro-inflammatory cytokines as IL-8, IL-6, iNOS, and TNF-α and scavenging ROS (Ferrini et al. 2022). Friedelin was found by Jiang et al. to reduce the development of tendinopathy by improving the rigidity of the Achilles tendon, reducing the infiltration of inflammatory cells, restoring the orderly arrangement of collagen fibers, and encouraging tendogenesis (Jiang et al. 2022). Sarfare and coauthors confirmed the in vitro anti-inflammatory impact of FRN via inhibiting both NF-κB and iNOS activity in LPS-induced macrophages (Sarfare et al. 2022). Nunes et al. showed that FRN reduced both NO and TNF release from macrophage exposed to LPS (Nunes et al. 2021). We suggested that the potential antioxidant and anti-inflammatory effects of FRN aid in the acceleration of wound repair. We also found that the faster healing procedures in FRN-treated groups may be attributed to the strongest VEGF and α-SMA immunoexpression in those groups. It is reported that α-SMA is a key marker for wound contraction and scar tissue formation (Putra et al. 2020).

## Conclusion

The present study proved the ability of FRN to downregulate both MRSA-virulence genes *seb* and *icaD*. Furthermore, the topical application of FRN significantly reduced the bacterial loads after 10 days of treatment and increased the rate of wound contraction. Histological examinations showed that infected mice treated with FRN had a complete re-epithelization and thick granulation tissue with regular collagen fiber deposition. Significant increases in VEGF and α-SMA expressions were observed in mice treated with FRN topically as reflected from immunohistochemistry results. Additionally, the relative expression levels of both *TNF-α* and *PGS-2* genes in groups treated with FRN were dose-dependently downregulated. We revealed that FRN is effective in the management of infected wounds, by inhibiting bacterial growth significantly and accelerating the wound repair process.

## Supplementary Information

Below is the link to the electronic supplementary material.

Supplementary file1 (DOCX 471 KB)

## Data Availability

All source data for this work (or generated in this study) are available upon reasonable request.

## Abbreviations

*ACTB*: Beta-actin
*FRN*: Friedelin
*IHC*: Immunohistochemical
*IL-6*: Interleukin-6
*IL-8*: Interleukin-8
*iNOS*: Inducible nitric oxide synthase
*IZ*: Inhibition zone
*LPS*: Lipopolysaccharides
*MIC*: Minimum inhibitory concentration
*MRSA*: Methicillin-resistant *Staphylococcus aureus*
*NF-κB*: Nuclear factor kappa B
*PGS-2*: Prostaglandin synthase-2
*ROS*: Reactive oxygen species
*TNF-α*: Tumor necrosis factor-α
*VAN*: Vancomycin
*VEGF*: Vascular endothelial growth factor
*α-SMA*: α-Smooth muscle act
