# Supplementary material for: Friedelin: A natural compound exhibited potent antibacterial, anti-inflammatory, and wound healing properties against MRSA-infected wounds
Source: Naunyn Schmiedebergs Arch Pharmacol. 2025 Mar 18;398(9):12103–13. doi: 10.1007/s00210-025-03965-8 (PMC12449404; doi:10.1007/s00210-025-03965-8)
Supplement: Supplementary file 1 — Supplementary file1 (DOCX 471 KB) [file 210_2025_3965_MOESM1_ESM.docx]

**Methods**

**Chemicals, drugs, and solvents**

Vancomycin® vials (500,000 IU) were purchased from Sigma-Tec (Sigma Pharmaceutical Industries, MO, USA). Carbopol 934 P was provided by Chemical Industries Development Company (Giza, Egypt). Triethanolamine was supplied from Morgan Chemicals IND. CO. (Cairo, Egypt). Standard commercial antibiotic discs, Vancomycin 30 µg and Amikacin 30 µg for Gram-positive bacteria were used as a positive control. All solvents used in the extraction and isolation procedures were supplied by PioChem. (Cairo, Egypt) and were of analytical grade.

**Plant extraction and identification**

**Plant material, extraction, fractionation, and isolation**

*Euphorbia grantii* Oliv. aerial parts were collected from El-Orman Botanic Garden, Giza, Egypt. The details of the isolation procedure was previously published, to obtain **compound 1** (110 mg) **(Fig. S1)**.

Ten kilograms of fresh plant material were dried to give 600 g of dried minced plant (powder). The air-dried plant material was extracted by methanol (5 L x 5) by maceration for 3 days. The collected extract was filtered and evaporated under reduced pressure to give 81.5 g of dry residue. A part of the total methanolic extract (50 g) was fractionated in methanol, water, and dichloromethane (DCM) with ratio 1:1:1, to yield 2 fractions; DCM, 30 g and the remaining mother liquor (ML), 22 g.

The DCM fraction (13 g) was fractionated by silica gel chromatography using *n-*hexane: ethyl acetate (EtOAc) as eluent to get 5 subfractions as follows; as follows; Fr.**1** (2.4 g), Fr. **2** (3 g), Fr. **3** (2 g), Fr. 4 (1.4 g), and Fr. **5** (eluted with 100% methanol, 1.8 g). Fr. 1 (2 g) was chromatographed with isocratic mobile phase *n-*hexane: EtOAc (95: 5) and 150 mL was collected in each subfractions. The same subfractions were collected to yield 3 main subfractions. Subfraction I weighed 350 mg which was re-chromatographed by MPLC column using isocratic elution of *n-*hexane: EtOAc (18.5:1.5), to obtain **compound 1** (5-13, 5 mL, 110 mg).

**Spectral data**

^1^H-NMR ppm (400 MHz, CDCL_3_) **(Table S1 and Fig. S2)**; 1.92, 1.67 (2H, *ddd*, H-1), 2.35, 2.26 (2H, *ddd*, H-2), 2.24 (1H, *q*, H-4), 1.70, 1.30 (2H, *m*, H-6), 1.49, 1.39 (2H, *m*, H-7), 1.38 (1H, *dd*, H-8), 1.54 (1H, *m*, H-10), 1.44, 1.26 (2H, *m,* H-11), 1.33, 1.32 (2H, *m*, H-12), 1.47, 1.27 (2H, *m*, H-15), 1.59, 1.18 (2H, *m*, H-16), 1.56 (1H, *m*, H-18), 1.37, 1.21 (2H, *m*, H-19), 1.51, 1.32 (2H, *m*, H-21), 1.51, 0.98 (2H, *m*, H-22), 0.88 (3H, *d*, H-23), 0.65 (3H, *s*, H-24), 0.82 (1H, *s*, H-25), 1.11 (1H, *s*, H-26), 1.14 (1H, *s*, H-27), 1.19 (1H, *s*, H-28), 1.16 (1H, *s*, H-29), 0.93(1H, *s*, H-30).

^13^C-NMR ppm (100 MHz, CDCL_3_) **(Table S1 and Fig. S3)**; 22.29 (C-1), 41.54 (C-2), 213.25 (C-3), 58.23 (C-4), 42.15 (C-5), 41.29 (C-6), 18.24 (C-7), 53.11 (C-8), 37.45(C-9), 59.48 (C-10), 35.63 (C-11), 30.51 (C-12), 39.70 (C-13), 38.30 (C-14), 32.42 (C-15), 36.01 (C-16), 30.00 (C-17), 42.80 (C-18), 35.35 (C-19), 28.17 (C-20), 32.77 (C-21), 39.26 (C-22), 6.83 (C-23), 14.66 (C-24), 17.95 (C-25), 20.26 (C-26), 18.67 (C-27), 32.09 (C-28), 35.03 (C-29), 31.79 (C-30).

**Molecular characterization of MRSA isolates**

The MRSA strain was isolated from the intensive care unit of a tertiary care hospital in Cairo, Egypt. All laboratory works were performed according to CLSI guidelines **[1]**. The isolated MRSA strain was previously identified phenotypically and genotypically **[2]**.

**Conventional and differential real-time PCR**

Primers used were supplied from Metabion (Germany) and sequences for the target genes were listed in **Table** **S2**. The details were mentioned in the supplementary file.

***In vitro* antimicrobial efficacy of friedelin (FRN)**

**Disc diffusion assay**

Antimicrobial activities of FRN were first screened for its inhibitory zone by the agar disc-diffusion method using Mueller-Hinton agar (Sigma-Aldrich, USA) media **[3].** The tests were performed in triplicates and repeated twice**.**

**Minimum inhibitory concentration (MIC)**

The minimum inhibitory concentration (MIC) was determined by the microbroth dilution method using Mueller-Hinton broth (Difco, USA). Different concentrations were prepared by two-fold serial dilution from 4096 µg /mL to 2 µg /mL. Positive control (VAN) was serially diluted from 64 to 0.12 µg /mL **[4, 5].**

**Gel formulation**

Sodium carboxymethyl cellulose (NaCMC, average molecular weight 250,000 g/mol) was purchased from Sigma-Aldrich Co., St. Louis, USA. Propylene glycol, 95% ethanol, and sodium benzoate were procured from El-Nasr Pharmaceuticals (Cairo, Egypt). Friedelin-loaded hydrogel was prepared under aseptic conditions by dispersing NaCMC (4% *w/v*) in propylene glycol in a porcelain dish. After that, the compound was added to the propylene glycol dispersion. In a beaker, sodium benzoate (1% *w/v*), which acted as a preservative, was dissolved in water. Then, the propylene glycol dispersion was added gradually to the beaker containing water and placed on a hotplate stirrer adjusted at 60 °C and 100 rpm. Finally, the gel was transferred into sterilized vials and stored in the refrigerator till further use.

***In vivo* animal model**

**Animals and wound induction**

35-adult male BALB/c mice (8-12 weeks old) with average weight 20-25 g were obtained from Holding Company of Biological Products and Vaccines (VACSERA), Helwan, Egypt. All mice were housed in plastic cages (3 or 4 mouse/ cage) in a well-ventilated environment and received a daily illumination of 12 hours of light. They were fed on dry commercial standard pellets and gained access to tap water *ad- libitum* throughout the experimental period. They were acclimatized to the environment for 2 weeks prior to the onset of the experiment to ensure their healthy state.

Prior to the wound induction, the mice were anesthetized with intramuscular injection of 50 mg/kg bwt Ketamine and 8 mg/kg bwt Xylazine. After that, the exposed skin area on the dorsal middle line of each mouse was shaved and cleaned with 70% ethanol, then the full-thickness circular wound (2 mm in diameter) was created using a sterile biopsy punch then separated individually (1 mouse/ cage). The MRSA-infected wound model was performed in all mice except the control non-infected group according to **[6]**. Within 5 min after wound induction, 10^9^ CFU MRSA was inoculated on the wound bed using small gauze. Treatment started locally for 10 consecutive days and involved the application of hydrogel was applied directly to the backs of mice wound formed on the back of the mouse twice a day.

Mice were randomly divided into 5 groups (n=7) and the topical treatment were given twice daily for 10 days as follows:

Group I: non-infected wound given Carpobol hydrogel.

Group II: MRSA-infected-wound given Carpobol hydrogel.

Group III: MRSA-infected-wound treated with 50 ppm VAN in Carpobol hydrogel.

Group IV: MRSA-infected-wound treated with 20 ppm FRN in Carpobol hydrogel **[7]**.

Group V: MRSA-infected-wound treated with 40 ppm FRN in Carpobol hydrogel **[7]**.

The progression of wound healing was monitored by measuring the size of wound at 0, 3, 7, 10 days post-excision using graded ruler to record the wound contraction as follows:

$$wound contraction=\frac{initial wound size-specific day wound size}{initial wound size}x 100$$

**Sampling**

After 10 days post-infection, all mice were humanly euthanized by cervical dislocation to collect specimens from the skin wound area. Some specimens were preserved in 10% neutral buffered formalin for 48 h to perform histopathological and immunohistochemical examination, while others kept at -80°c for few minutes to perform the bacteriological and molecular studies.

**Re-isolation and quantification of bacteria in the wound bed**

To measure the viable bacterial cells within the wound, a 4 mm disposable skin biopsy punch (Acutderm Inc., Fort Lauderdale, FL) was used to remove a disc of wound bed material. The disc was placed in 1.0 ml of sterile PBS in a stomacher bag and manually disrupted. Serial 10-fold dilutions of homogenate were plated via spiral plater (Autoplate; Advanced Instruments, Inc., Norwood, MA) onto eosin methylene blue (EMB) agar (Becton, Dickinson and Co., Sparks, MD). Plates were incubated overnight at 37°C, and then CFU were enumerated.

**Conventional and differential real-time PCR**

The DNA were isolated from all isolates using Gene jet Genomic DNA Purification Kit (Thermo Scientific, EU) following the manufacturer’s recommendations. The PCR reaction was set as follows:1 µL of each primer were mixed with 12.5 µL Emerald Amp Max PCR Master Mix (Takara, Japan), and 5 µL DNA template in an Applied biosystem 9700 thermocyclers (Thermo Fisher). The products of PCR were separated by electrophoresis on 1.5% agarose gel (Applichem, Germany, GmbH) in 1x TBE buffer at room temperature. The gel was then photographed by a gel documentation system (Alpha Innotech, Biometra) and the data was analyzed through computer software. The total RNA extraction was performed following the “Enzymatic Lysis” procedure of QIAamp RNeasy Mini kit (Qiagen, Germany, GmbH). The primer sequences for the target genes were collected in **Table S2**. For preparation of the real time PCR mixture, QuantiTect SYBR Green PCR Master Mix (Qiagen, Germany, GmbH) and RevertAid Reverse Transcriptase (200 U/L) (Thermo Fisher) were used then the reaction was carried out in BioRad thermocycler with the following thermal cycling steps: an initial denaturation at 94°C for 3 min, followed by 40 cycles of (denaturation at 94°C for 30 s, annealing at 57°C for 30s, and extension at 72°C for 30 s). The amplicons were electrophoresed on 1.5% agarose gel. The fold change in the expression was calculated using the 2^-ΔΔCt^ method.

**Quantitative RT-PCR analysis of PGS-2 and TNF-α genes**

Using the Qiagen RNeasy kit (Qiagen AB, Hilden, Germany), the total RNA was extracted and purified in accordance with the manufacturer's instructions. Then, according with the manufacturer's instructions to convert the isolated RNA to cDNA. The quantitative PCR assays for TNF-α and PTGS-2 were designed and validated **(Table S3)**. The Primer3 website (<https://primer3.ut.ee/>) was used to design the used primer sets. The details of the method were previously published **[8].** Relative gene expression levels were calculated by normalizing gene expression of each gene using beta actin gene and the ΔΔCt method **[9].**

**Histopathology**

Formalin fixed skin tissue samples were processed by the conventional method using ascending grade of alcohol and xylene following method of **[10].** After that, samples were imbedded in paraffin wax and sliced in to 4.5 μm sections. All sections were stained by hematoxylin and eosin (H&E) and examined under BX43 light Olympus microscope (Olympus Corporation, Tokyo, Japan) to evaluate the extent of wound healing in different experimental groups. Images were captured by Olympus DP27 digital camera (Olympus Corporation, Tokyo, Japan) attached to cell sense dimension software.

The extent of wound healing in all sections were qualitatively evaluated using classical semi-quantitative scoring system according to the method portrayed by **[11].** All stages of wound healing include, Re-epithelization, vascular congestion, exudation, hemorrhage, inflammatory cells infiltration, angiogenesis, granulation tissue formation, and collagen organization were blindly graded in all sections. Based on the progression of those criteria, a four-point grading scale was used as follows: (-) = zero (non), (+) = <25% (slight), (++) = 25%:50% (moderate), (+++) = 50-75% (marked), and (++++) = > 75% (extensive sever).

**Immunohistochemistry**

The deparaffinized and dehydrated skin tissue sections were washed in PBS-T, microwaved for antigen retrieval, and blocked in 1% bovine serum albumin. Afterwards, sections were incubated with primary antibody against vascular endothelial growth factor (VEGF) and alpha smooth muscle actin (α-SMA) (Abcam, Cambridge, UK) at 1:200 dilutions overnight. Lately, they were washed and incubated with reagents included in antigen detection system (Power‐Stain 1.0 Poly HRP DAP Kit; Sakura). Finally, sections were counterstained by hematoxylin, mounted by a DPX mounting medium and then examined under a light microscope to evaluate the severity of immunoexpression.

The immunohistochemical (IHC) results were analyzed using a combination of quantitative and qualitative methods. The relative percentage of immunopositively cells were calculated in relation to the total number of target cells using Image J software. Afterwards, the qualitative results were categorized in six-pointed grading scale as the following: (1) 0–5%, (2) 5–25%, (3) 25–50%, (4) 50–75%, (5) 75–95%, (6) >95%). Additionally, we assessed the intensity of immunostaining for each marker in various groups. The intensity is categorized in four-pointed grading scale as the following: (0, negative; 1, weak positive; 2, moderate positive; and 3, strong positive). The final score is calculated by adding each score  **[12].**

**Statistical analysis**

All parametric values were shown as means ± standard error of mean (SEM). Results were analyzed using one-way ANOVA and post-hoc Duncan's test in SPSS version 20. P values < 0.05 indicate statistical significance. The immunohistochemistry score was expressed as median ± IQR and analyzed using the Kruskal-Wallis H test and Mann-Whitney U test.

**Fig. S1.** The chemical structure of friedelin.

**Fig. S2.** ^1^H-NMR of friedelin.

**Fig. S3.** ^13^C-NMR of friedelin.

**
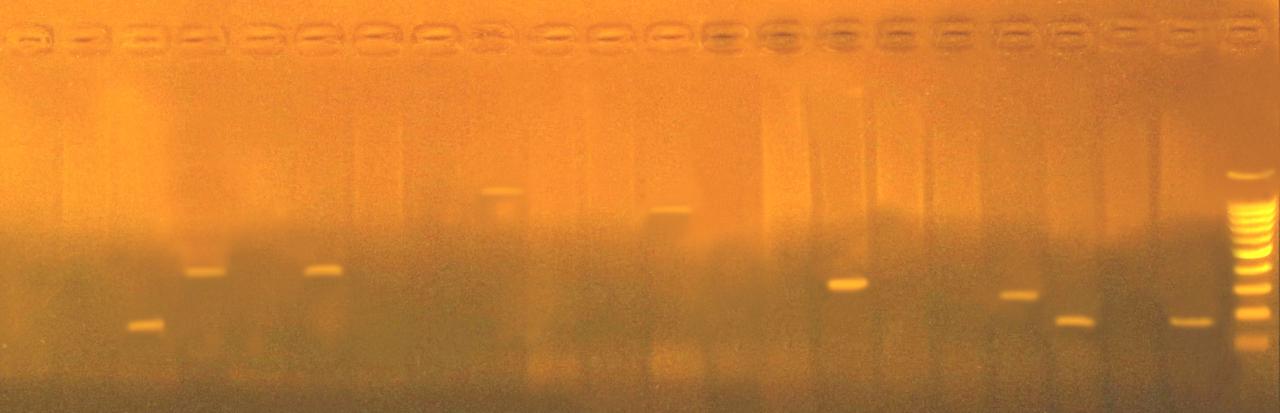
**


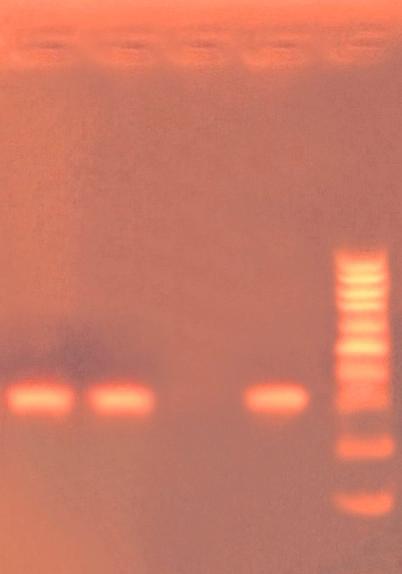


**Fig. S4.** Uncropped Gels image.

**Table S1.** ^1^H-NMR and ^13^C-NMR data of friedelin as compared to reference data.

| **^1^H-NMR** | | | **^13^C-NMR** | |
| --- | --- | --- | --- | --- |
| No. | *δ*Φ | *δ***#** | *δ*Φ | *δ***#** |
| 1 | 1.92, 1.67  (2H,ddd) | 1.95, 1.71 (2H,ddd) | 22.29 | 22.3 |
| 2 | 2.35, 2.26  (2H,ddd) | 2.37, 2.27  (2H,ddd) | 41.54 | 41.5 |
| 3 | - | - | 213.25 | 213.2 |
| 4 | 2.24 (1H,q) | 2.25 (1H,q) | 58.23 | 58.2 |
| 5 | - | - | 42.15 | 42.1 |
| 6 | 1.70, 1.30  (2H, m) | 1.74,1.28  (2H, m) | 41.29 | 41.3 |
| 7 | 1.49, 1.39  (2H, m) | 1.49, 1.36  (2H, m) | 18.24 | 18.2 |
| 8 | 1.38  (1H, dd) | 1.38  (1H, dd) | 53.11 | 53.1 |
| 9 | - | - | 37.45 | 37.4 |
| 10 | 1.54 (1H,m) | 1.53(1H,m) | 59.48 | 59.5 |
| 11 | 1.44, 1.26  (2H,m) | 1.45, 1.26  (2H,m) | 35.63 | 35.6 |
| 12 | 1.33, 1.32  (2H,m) | 1.33, 1.32  (2H,m) | 30.51 | 30.5 |
| 13 | - | - | 39.70 | 39.7 |
| 14 | - | - | 38.30 | 38.3 |
| 15 | 1.47, 1.27  (2H,m) | 1.47,1.27  (2H,m) | 32.42 | 32.4 |
| 16 | 1.59, 1.18 | 1.58,1.35  (2H,m) | 36.01 | 36.0 |
| 17 | - | - | 30.00 | 30.0 |
| 18 | 1.56 (1H,m) | 1.56(1H,m) | 42.80 | 42.8 |
| 19 | 1.37, 1.21  (2H,m) | 1.37,1.22  (2H,m) | 35.35 | 35.3 |
| 20 | - | - | 28.17 | 28.2 |
| 21 | 1.51, 1.32  (2H, m) | 1.50,1.31  (2H, m) | 32.77 | 32.8 |
| 22 | 1.51, 0.98  (2H, m) | 1.51, 0.95  (2H, m) | 39.26 | 39.2 |
| 23 | 0.88 (3H, d) | 0.88 (3H, d) | 6.83 | 7.0 |
| 24 | 0.65 (3H, s) | 0.73 (3H, s) | 14.66 | 14.6 |
| 25 | 0.82 | 0.87 | 17.95 | 17.9 |
| 26 | 1.11 | 1.01 | 20.26 | 20.2 |
| 27 | 1.14 | 1.05 | 18.67 | 18.6 |
| 28 | 1.19 | 1.18 | 32.09 | 32.1 |
| 29 | 1.16 | 1.00 | 35.03 | 35.0 |
| 30 | 0.93 | 0.94 | 31.79 | 31.8 |

Φ → isolated compounds, # → data from literature [13].

**Table S2. Primers sequences of the target genes of MRSA isolates**

| **Target gene** | **Primers sequences** | **Amplified segment (bp)** | **Reference** |
| --- | --- | --- | --- |
|  |  |  |  |
| ***mecA*** | GTA GAA ATG ACT GAA CGT CCG ATA A | 310 | **[14]** |
|  | CCA ATT CCA CAT TGT TTC GGT CTA A |  |  |
| ***Seb*** | GTATGGTGGTGTAACTGAGC | 164 | **[15]** |
|  | CCAAATAGTGACGAGTTAGG |  |  |
| ***Sed*** | CCAATAATAGGAGAAAATAAAAG | 278 |  |
|  | ATTGGTATTTTTTTTCGTTC |  |  |
| ***Tsst*** | ACCCCTGTTCCCTTATCATC | 326 |  |
|  | TTTTCAGTATTTGTAACGCC |  |  |
| ***Hlg*** | GCCAATCCGTTATTAGAAAATGC | 937 | **[16]** |
|  | CCATAGACGTAGCAACGGAT |  |  |
| ***icaA*** | CCT AAC TAA CGA AAG GTA G | 1315 | **[17]** |
|  | AAG ATA TAG CGATAA GTG C |  |  |
| ***icaD*** | AAA CGTAAG AGA GGT GG | 381 |  |
|  | GGC AAT ATG ATC AAGATA |  |  |
| ***fnbA*** | CATAAATTGGGAGCAGCATCA | 127 | **[18]** |
|  | ATCAGCAGCTGAATTCCCATT |  |  |

**Abbreviations: *fnbA*,** Fibronectin-binding protein A; ***icaD****,* Inhibitor of caspase-activated deoxyribonuclease; ***icaA*,** Intercellular adhesion A; ***Hlg*,** gamma-hemolysin; ***Tsst*,** Toxic shock syndrome toxin-1; ***Sed*,** staphylococcal enterotoxin D; and ***Seb*,** staphylococcal enterotoxin B.

**Table S3. The primer sets of the studied genes.**

| **Gene symbol** | **Gene description** | **Accession number** | **Forward Primer Sequence** | **Reverse Primer Sequence** |
| --- | --- | --- | --- | --- |
| *TNF-α* | Tumor necrosis factor | [NM_013693.3](https://www.ncbi.nlm.nih.gov/entrez/viewer.fcgi?db=nucleotide&id=518831586) | TGTAGCCCACGTCGTAGCAA | ATAGCAAATCGGCTGACGGT |
| *PGS-2* | prostaglandin-endoperoxide synthase 2 | [NM_011198.5](https://www.ncbi.nlm.nih.gov/entrez/viewer.fcgi?db=nucleotide&id=2316037418) | CATCCCCTTCCTGCGAAGTT | CATGGGAGTTGGGCAGTCAT |
| *ACTB* | Beta actin | [NM_007393.5](https://www.ncbi.nlm.nih.gov/entrez/viewer.fcgi?db=nucleotide&id=930945786) | CCACCATGTACCCAGGCATT | AGGGTGTAAAACGCAGCTCA |

**References**

[1] Cockerill FR, Wikler M, Bush K, Dudley M, Eliopoulos G, Hardy D. Clinical and laboratory standards institute. Performance standards for antimicrobial susceptibility testing: twenty-second informational supplement 2012.

[2] Rana E, Rania AK, Hamdallah Z, Alaa E-DSH, Tarek HE. Study on prevalence and genetic discrimination of methicillin-resistant Staphylococcus aureus (MRSA) in Egyptian hospitals. African Journal of Microbiology Research 2018;12:629-46.

[3] Bauer A, Kirby W, Sherris JC, Turck M. Antibiotic susceptibility testing by a standardized single disk method. American journal of clinical pathology 1966;45:493-6.

[4] Caldwell MD. Bacteria and antibiotics in wound healing. Surgical Clinics 2020;100:757-76.

[5] Wiegand I, Hilpert K, Hancock RE. Agar and broth dilution methods to determine the minimal inhibitory concentration (MIC) of antimicrobial substances. Nature protocols 2008;3:163-75.

[6] El-Gayar MH, Aboshanab KM, Aboulwafa MM, Hassouna NA. Antivirulence and wound healing effects of royal jelly and garlic extract for the control of MRSA skin infections. Wound Medicine 2016;13:18-27.

[7] Antonisamy P, Duraipandiyan V, Ignacimuthu S. Anti-inflammatory, analgesic and antipyretic effects of friedelin isolated from Azima tetracantha Lam. in mouse and rat models. Journal of pharmacy and pharmacology 2011;63:1070-7.

[8] Farid MF, Abouelela YS, Yasin NA, Al-Mokaddem AK, Prince A, Ibrahim MA, et al. Laser-activated autologous adipose tissue-derived stromal vascular fraction restores spinal cord architecture and function in multiple sclerosis cat model. Stem Cell Research & Therapy 2023;14:1-16.

[9] Livak KJ, Schmittgen TD. Analysis of relative gene expression data using real-time quantitative PCR and the 2− ΔΔCT method. methods 2001;25:402-8.

[10] Bancroft JD, Gamble M. Theory and practice of histological techniques: Elsevier health sciences; 2008.

[11] Khalaf AA, Hassanen EI, Zaki AR, Tohamy AF, Ibrahim MA. Histopathological, immunohistochemical, and molecular studies for determination of wound age and vitality in rats. International Wound Journal 2019;16:1416-25.

[12] Tzankov A, Zlobec I, Went P, Robl H, Hoeller S, Dirnhofer S. Prognostic immunophenotypic biomarker studies in diffuse large B cell lymphoma with special emphasis on rational determination of cut-off scores. Leukemia & lymphoma 2010;51:199-212.

[13] Mann A, Ibrahim K, Oyewale AO, Amupitan JO, Fatope MO, Okogun JIJAJoC. Antimycobacterial friedelane-terpenoid from the root bark of Terminalia avicennioides. 2011;1:52-5.

[14] McClure J-A, Conly JM, Lau V, Elsayed S, Louie T, Hutchins W, et al. Novel multiplex PCR assay for detection of the staphylococcal virulence marker Panton-Valentine leukocidin genes and simultaneous discrimination of methicillin-susceptible from-resistant staphylococci. Journal of clinical microbiology 2006;44:1141-4.

[15] Mehrotra M, Wang G, Johnson WM. Multiplex PCR for detection of genes for Staphylococcus aureus enterotoxins, exfoliative toxins, toxic shock syndrome toxin 1, and methicillin resistance. Journal of clinical microbiology 2000;38:1032-5.

[16] Kumar JD, Negi YK, Gaur A, Khanna D. Detection of virulence genes in Staphylococcus aureus isolated from paper currency. International Journal of Infectious Diseases 2009;13:e450-e5.

[17] Ciftci A, Findik A, Onuk EE, Savasan S. Detection of methicillin resistance and slime factor production of Staphylococcus aureus in bovine mastitis. Brazilian Journal of Microbiology 2009;40:254-61.

[18] Vancraeynest D, Hermans K, Haesebrouck F. Genotypic and phenotypic screening of high and low virulence Staphylococcus aureus isolates from rabbits for biofilm formation and MSCRAMMs. Veterinary microbiology 2004;103:241-7.
